# Methylation mediated silencing of *TMS1/ASC *gene in prostate cancer

**DOI:** 10.1186/1476-4598-5-28

**Published:** 2006-07-18

**Authors:** Partha M Das, Kavitha Ramachandran, Jane VanWert, Larry Ferdinand, Gopal Gopisetty, Isildinha M Reis, Rakesh Singal

**Affiliations:** 1 Sylvester Comprehensive Cancer Center, University of Miami, Miami, FL – 33136, USA; 2 Miami VA Medical Center, Miami, FL-33136, USA

## Abstract

**Background:**

Transcriptional silencing associated with aberrant promoter methylation has been established as an alternate pathway for the development of cancer by inactivating tumor suppressor genes. *TMS1 *(Target of Methylation induced Silencing), also known as *ASC *(Apoptosis Speck like protein containing a CARD) is a tumor suppressor gene which encodes for a CARD (caspase recruitment domain) containing regulatory protein and has been shown to promote apoptosis directly and by activation of downstream caspases. This study describes the methylation induced silencing of *TMS1/ASC *gene in prostate cancer cell lines. We also examined the prevalence of *TMS1/ASC *gene methylation in prostate cancer tissue samples in an effort to correlate race and clinico-pathological features with *TMS1/ASC *gene methylation.

**Results:**

Loss of *TMS1/ASC *gene expression associated with complete methylation of the promoter region was observed in LNCaP cells. Gene expression was restored by a demethylating agent, 5-aza-2'deoxycytidine, but not by a histone deacetylase inhibitor, Trichostatin A. Chromatin Immunoprecipitation (ChIP) assay showed enrichment of MBD3 (methyl binding domain protein 3) to a higher degree than commonly associated MBDs and MeCP2. We evaluated the methylation pattern in 66 prostate cancer and 34 benign prostatic hyperplasia tissue samples. *TMS1/ASC *gene methylation was more prevalent in prostate cancer cases than controls in White patients (OR 7.6, p 0.002) while no difference between the cases and controls was seen in Black patients (OR 1.1, p 0.91).

**Conclusion:**

Our study demonstrates that methylation-mediated silencing of *TMS1/ASC *is a frequent event in prostate cancer, thus identifying a new potential diagnostic and prognostic marker for the treatment of the disease. Racial differences in *TMS1/ASC *methylation patterns implicate the probable role of molecular markers in determining in susceptibility to prostate cancer in different ethnic groups.

## Background

Gene silencing associated with aberrant promoter methylation has been suggested as an alternate pathway for development of cancer [1]. This form of epigenetic change contributes to tumor initiation and progression by transcriptional silencing of tumor-suppressor genes. Several genes have been shown to be epigenetically inactivated in a wide range of tumors [2] and most neoplasms show hypermethylation of one or more genes [3-5]. This has led to the concept of a 'hypermethylation profile' of tumors which could have potential clinical applications [5-7]. The hypermethylated genes can be broadly classified as those involved in cell cycle regulation (p16INK4a, p15, Rb), genes associated with DNA repair (BRCA1, MGMT), apoptosis (DAPK), and drug detoxification (GSTPi), drug resistance (MGMT), cellular differentiation, angiogenesis (THBS1) and metastasis (E-cadherin) [2].

Epigenetic dysregulation of an apoptotic pathway appears to be connected to the development of many cancers as confirmed by several research communications [8,9]. Apoptosis or 'programmed cell death' is essential for embryonic development and also plays an important role in the immune system and maintenance of cellular homeostasis [10]. A defect in apoptosis is implicated in neurodegenerative diseases, autoimmunity, and cancer and in chemo-resistance. Multiple genes direct the apoptotic pathway to restrain the inappropriate proliferation of cells and a defect in the signaling mechanism gives the cancer cells an added survival advantage leading to tumor initiation, progression and even drug resistance. Apoptosis is mediated by a family of cystine proteases called caspases. There are two groups of caspases – the initiator caspases (CASP8, CASP9 and CASP10) and the effector caspases (CASP3, CASP6 and CASP7). Caspases exist as latent pro-enzymes and are activated by proteolytic cleavage. Several key genes involved in apoptosis have been showed to be target of epigenetic changes [9].

Recently, *TMS1 *was shown to be aberrantly hypermethylated in breast cancer tissues and cell lines [11]. This gene is also known as ASC (Apoptosis Speck Like protein containing a CARD) [12]. *TMS1/ASC *encodes a 22-kDa CARD protein and promotes apoptosis in a caspase 9 dependent pathway. *TMS1/ASC *has been shown to bind with various proteins like Pyrin, Ipaf and copyrin/PYPAF1[12]. In addition to its role in cancer development there is ample evidence which suggests that *TMS1/ASC *is involved in immune responses and NF-κB and caspase1 activation [13,14]. The downregulation of *TMS1/ASC *in breast cancer cell lines correlates with dense methylation on the CpG islands. Methylation of the promoter region of *TMS1/ASC *has also been identified in small cell lung cancer and non-small cell lung cancer [15], human glioblastoma [16], ovarian tumors [17,18], colorectal cancer [19], neuroblastoma [20], and melanoma [21]. However in a study by Roman-Gomez et. al., on acute lymphoblastic leukemia patients no correlation was found with methylation of the *TMS1/ASC *gene [22].

So far no studies have been reported on the role of *TMS1/ASC *in prostate cancer. In this study we examined the mechanism of *TMS1/ASC *gene silencing in prostate cancer cell lines. We also studied the prevalence of *TMS1/ASC *gene methylation in prostate cancer tissue samples as well as the association of race and clinico-pathological features with *TMS1/ASC *gene methylation.

## Results

### Expression and methylation status of TMS1/ASC gene in prostate cancer cell lines

**Figure 1:**
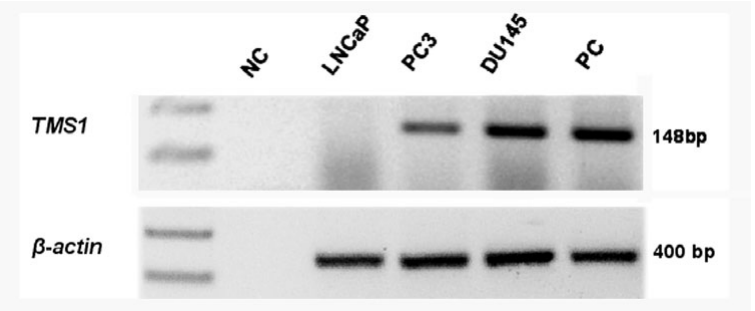
Expression pattern of *TMS1/ASC *gene. Reverse transcription PCR analysis showing expression of *TMS1/ASC *gene in prostate cancer cell lines. β-actin was used as a control for RNA integrity. **NC**-negative control, **PC**-positive control.

By RTPCR (reverse transcriptase polymerase chain reaction), we first analyzed the expression status of *TMS1/ASC *in three prostate cancer cell lines – LNCaP, PC3 and DU145 by RT-PCR. LNCaP exhibited complete loss of *TMS1/ASC *transcript whereas partial expression was detected in the remaining two cell lines (Fig 1). β-actin was used as control for RNA integrity and loading.

**Figure 2:**
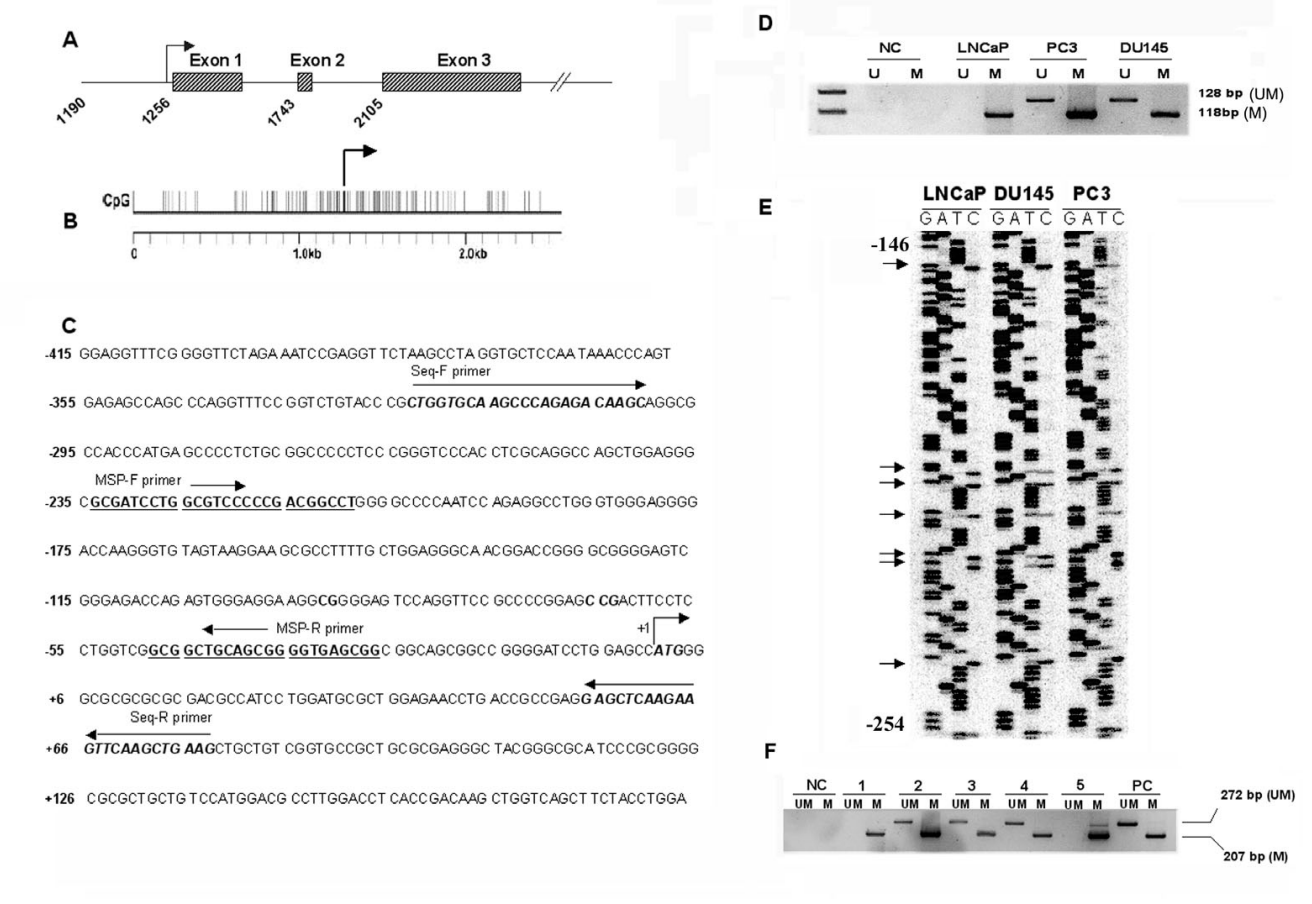
Methylation pattern of *TMS1/ASC*. *Panel A *– graphical representation of the structure of *TMS1/ASC *gene showing position of the three exons. *Panel B *shows the location of the CpG dinucleotides. *Panel C *shows the postion of the primers used for MS-PCR and bisulfite sequencing in the promoter region of *TMS1/ASC*. Positions are indicated relative to translation start site. *Panel D *shows MS-PCR analysis of *TMS1/ASC *gene on the different prostate cancer cell lines. LNCaP exhibits complete methylation of *TMS1/ASC *gene. PC3 and Du145 have both the methylated as well as the unmethylated allele and are consequently expressed. **NC**-negative control, **U**-unmethylated allele, **M**-methylated allele. The methylation pattern correlates with the bisulfite genomic sequencing shown on *panel E*. Completely methylated cytosines (black arrow) in LNCaP are not converted to thymidine following bisulfite treatment and show up on the C lane whereas partially methylated cytosines in the CpG's are seen both in the T lane as well as in the C lane in PC3 and Du145 cell lines. Positions are indicated relative of the translation start site. Panel F shows representative examples of 5 prostate cancer tissue samples analyzed by MS-PCR and gel electrophoresis. Presence of a band in lanes marked as **UM **indicates presence of unmethylated allele and a band in the lanes marked **M **denotes a methylated allele; **NC**-negative control; **PC**-positive control.

Methylation-mediated deregulation of *TMS1/ASC *has been described in breast cancer and in other tumors. To determine if methylation was responsible for down regulation of *TMS1/ASC *in prostate cancer cell lines, we performed MS-PCR (methylation specific PCR) using both methylated and unmethylated primers (Fig 2C). Complete methylation of the promoter region was seen in LNCaP cells whereas PC3 and DU145 showed partial methylation of the *TMS1/ASC *gene (Fig 2D). *TMS1/ASC *expression inversely correlated with the methylation status. To confirm our MS-PCR findings we performed bisulfite genomic sequencing of the sodium bisulfite modified DNA in the three cell lines. In LNCaP cell line the cytosines in the non-CpG sites were converted to thymidine but the cytosines associated with CpG islands remained unmodified after bisulfite treatment thereby demonstrating complete methylation. PC3 and DU145 showed a mixed pattern of methylated as well as unmethylated cytosine in the CpG dinucleotides (Fig 2E). We also performed MS-PCR on DNA extracted from 5 representative tissue samples of prostate cancer patients. Two of the samples showed complete methylation, while the remaining three showed partial methylation of *TMS1/ASC *gene promoter (Fig 2F).

### 5-Aza-2'-deoxycytidine and TSA treatment of LNCaP cell line

**Figure 3:**
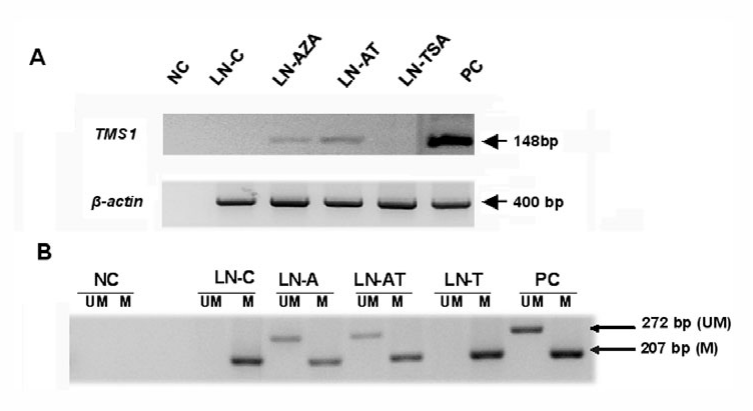
Expression and methylation pattern of *TMS1/ASC *gene in drug treated LNCaP cells and in Tumor tissues. *Panel A *shows restoration of *TMS1/ASC *gene expression following treatment with 5-Aza but not with TSA alone. *Panel B *– MS-PCR analysis on the drug treated LNCaP cells confirm expression of *TMS1/ASC *is associated with partial demethylation of the gene seen as a band on the UM lane in 5-Aza treated cells. Presence of a band in lanes marked as **UM **indicates presence of unmethylated allele and a band in the lanes marked **M **denotes a methylated allele. **NC**-negative control; **PC**-positive control; **UM**-unmethylated allele; **M**-methylated allele; **LN-C**-untreated LNCaP cells; **LN-AZA **-5-AZA-2'-deoxycytidine treated cells; **LN-AT **– 5-AZA-2'-deoxycytidine followed by TSA; **LN-TSA **– TSA treated cells.

We treated LNCaP cells with 5-Aza-2'-deoxycytidine in an attempt to induce *TMS1/ASC *gene expression. 5-Aza-2'-deoxycytidine is a demethylating agent and forms a covalent complex with DNA methyltransferase thus inhibiting DNA methylation. The *TMS1/ASC *gene expression was examined by RT-PCR analysis and methylation status of *TMS1/ASC *gene examined by MS-PCR of the bisulfite modified drug treated DNA. Following 5-Aza-2'-deoxycytidine treatment the *TMS1/ASC *gene underwent partial demethylation and resulted in expression of the gene (Fig 3). Since methylated DNA binds to methylcytosine binding proteins which in turn interact with histone deacetylases (HDAC), we treated the LNCaP cells with a histone deacetylase inhibitor TSA to see if it can induce expression of the *TMS1/ASC *gene. TSA treatment failed to induce *TMS1/ASC *mRNA expression in LNCaP cells (Fig 3A). Methylation pattern analysis of drug treated cells showed that *TMS1/ASC *promoter remained fully methylated in TSA-treated cells whereas both unmethylated and methylated alleles exist in 5-AZA-2'-deoxycytidine treated cells (Fig. 3B).

### Histone acetylation and interaction of methylcytosine binding proteins with TMS1/ASC promoter in LNCaP cell line

**Figure 4:**
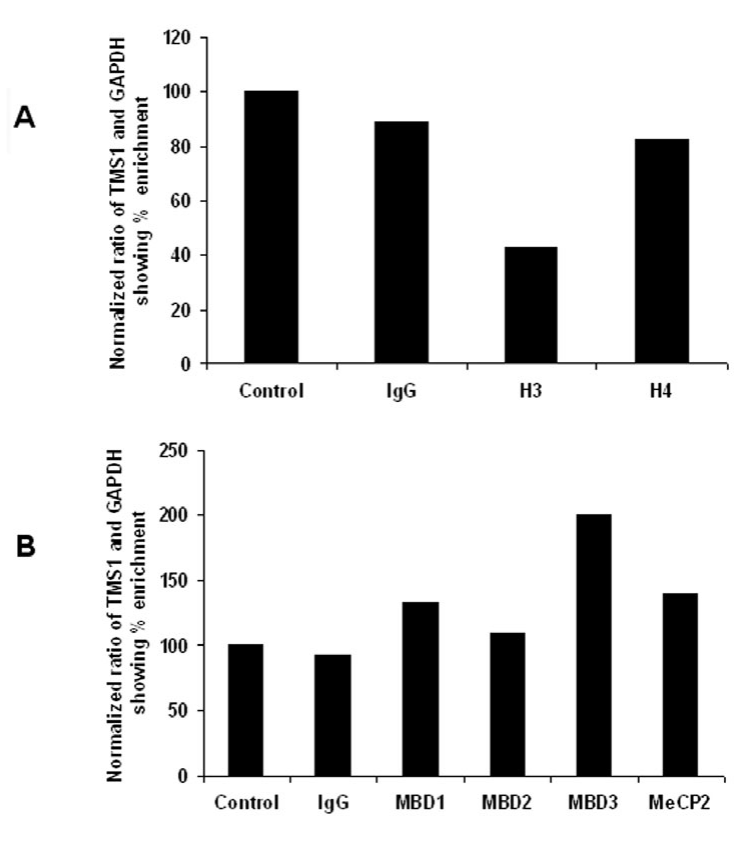
Binding pattern of acetylated histones (*panel A*) and MBD's (*panel B*) to the methylated *TMS1/ASC *gene. Chromatin immunoprecipitation performed on formalin fixed and sonicated LNCaP chromatin shows enrichment of MBD3 in the immunoprecipitated DNA and low levels of acetylated H3 suggesting deacetylation of H3 in the silenced TMS1/ASC gene.

Recent studies have shown that deacetylation of histone H3 and H4 by the HDAC's presumably leads to the formation of a chromatin environment that inhibits transcription. To determine if methylation induced silencing of *TMS1/ASC *is associated with changes in histone acetylation pattern we studied the pattern of histone acetylation at the *TMS1/ASC *promoter in LNCaP cells using ChIP assay. Neither acetylated H3 nor acetylated H4 binds to *TMS1/ASC *promoter region in LNCaP (Fig 4A). Since methylcytosine binding proteins mediate the repressive effect of methylated DNA, we examined the interaction of methylcytosine binding proteins with the *TMS1/ASC *promoter. The TMS1 promoter interacted mainly with MBD3 though minimal binding of MBD1, MBD2 and MeCP2 at *TMS1/ASC *promoter was also seen (Fig 4B).

### Patient characteristics and association with TMS1/ASC gene methylation

Table 2 shows the demographics and clinical characteristics of the study subjects. The prostate cancer patients (51 – 80 yrs) were 7 years younger than BPH (53–88 yrs) on an average. However the racial distribution was similar. Most of the patients were stage 2–3 and had Gleason score of 6–7. Table 3 shows the frequency of *TMS1/ASC *gene methylation in all the samples examined. *TMS1/ASC *gene methylation was seen in 63.6% of prostate cancer but in only 35.3% of BPH patients with an age-adjusted odds ratio of 3.7 (95% CI 1.4 – 9.4). The prevalence of *TMS1/ASC *gene methylation in prostate cancer patients was found to be similar in both races (66.7% in Blacks as compared to 62.2% in Whites). However comparing the prevalence of *TMS1/ASC *methylation between prostate-cancer patients and controls, there was no difference among Blacks (prevalence 66.7% for patients and 58.3% for controls), whereas in Whites a significant difference was observed (prevalence 62.2% for prostate cancer cases and 22.7% for BPH controls). The age adjusted odds ratio comparing cases to controls among White patients was 7.6 (p = 0.002). We also studied the effect of age and *TMS1/ASC *gene methylation but no association with early or late onset prostate cancer was observed (data not shown). No association with stage of the disease was observed but there was a tendency of *TMS1/ASC *gene methylation to be more frequent in tumors having Gleason score 7 or higher. (Table 4)

**Table 1:** Table showing details of primers used and cycling conditions for MS-PCR, RT-PCR and PCR amplification of the CHIP DNA.

***Gene***	***Primer sequence (5'-3')***	***Anneal. Temp. (°C)***	***No. of Cycles***
***TMS1/ASC methylated***	F- CGA TTT TGG CGT TTT TCG ACG GTT	65	35
	R- CCG CTC ACC CCG CTA CAA CCG C		
***TMS1/ASC Unmethylated***	F- TTG TTG GAG GGT AAT GGA TT	58	35
	R- CCC ACA AAA ATA CAC CCA TA		
***TMS1/ASC RT-PCR***	F- GGA CGC CTT GGC CCT CAC CG	65	35
	R- GGC GCG GCT CCA GAG CCC TG		
***TMS1/ASC CHIP***	F- GAG TCG GGA GAC CAG AGT GGA	68	50
	R- ACA GCA GCT TCA GCTT GAA CTT CTT G		
***β-actin***	F- ACC ATG GAT GAT GAT ATC GC	60	30
	R- ACA GGC TGG GGT GTT GAA G		
***GAPDH***	F- CCC CAC ACA CAT GCA CTT ACC	65	50
	R- CCT AGT CCC AGG GCT TTG ATT		

**Table 2:** Characteristics of 66 prostate-cancer patients and 34 BPH controls.

	**Prostate cancer**		**Control (BPH)**		
	**n**	**%**	**n**	**%**	**P-value**
**Race**					
Black	21	31.8	12	35.3	0.82
White	45	68.2	22	64.7	
**Stage**					
II	42	63.6			
III	19	28.8			
IV	4	6.1			
Unknown	1	1.5			
**Gleason Score**					
5	3	4.6			
6	26	39.4			
7	32	48.4			
8	3	4.6			
9	2	3.0			
**Preoperative serum PSA**					
< 4	5	7.6			
4.0 – 8	46	69.7			
8.1 – 12	8	12.1			
> 12	7	10.6			
Mean age (range)	64 yrs (51 – 80)		71 yrs (53 – 88)		< 0.001

**Table 3:** Overall and race-specific frequencies of *TMS1/ASC *gene methylation in prostate-cancer patients and BPH controls, with age-adjusted odds-ratio estimates of the relative risk of prostate cancer associated with *TMS1/ASC *gene methylation.

	**Number and (%) of tissues with *TMS1/ASC *methylation**		Age-adjusted odds ratio* (95% CI)	
**Patients**	**Prostate Cancer**	**Control (BPH)**		**P-value**
**All**	42/66 (63.6)	12/34 (35.3)	3.7 (1.4 – 9.4)	0.008
**Blacks**	14/21 (66.7)	7/12 (58.3)	1.1 (0.2 – 5.5)	0.91
**Whites **	28/45 (62.2)	5/22 (22.7)	7.6 (2.1 – 27.3)	0.002

* For the overall odds ratio estimate, we modeled the logit of the probability of being a case (p), as a function of *TMS1/ASC *gene methylation and age: ln [p/(1-p)] = b_0 _+ b_1 _× *TMS1/ASC *+ b_2 _× Age. For the race-specific age-adjusted odds-ratio estimates, the following model was fitted: ln [p/(1-p)] = b_0 _+ b_1 _× *TMS1/ASC *+ b_2 _× Age + b_3 _× Race + b_4 _× *TMS1/ASC *× Race. The interaction term TMS1/ASC*Race was marginally significant (p = 0.068).

**Table 4:** Effect of *TMS1/ASC *gene methylation on clinical stage (II or III), and Gleason score (6 or 7).

	**Number and (%) of tissues with TMS1/ASC methylation**	Age-adjusted odds-ratio* (95% CI)	**P-value**
**Clinical stage**			
II (reference)	28/42 (66.7)		
III	11/19 (57.9)	0.7 (0.2 – 2.1)	0.53
**Gleason score**			
6 (reference)	14/26 (53.9)		
7	23/32 (71.9)	2.3 (0.7 – 7.2)	0.15

* Age-adjusted relative risk estimates of stage III vs. II, or Gleason score 7 vs. 6, derived from logistic model ln (p/q) = b_0 _+ b_1 _× *TMS1/ASC *+ b_2 _× Age, where p is either the probability of having stage III disease or of having Gleason score 7, and q = (1-p) is the probability of the other category.

## Discussion

Our study shows a clear correlation between *TMS1/ASC *methylation and silencing of the gene suggesting a role in prostate cancer cell lines. Re-expression accompanied by partial demethylation of *TMS1/ASC *following 5-AZA-2'-deoxycytidine confirms that methylation is responsible for transcriptional silencing of this gene. Lack of response to TSA treatment suggests that histone acetylation does not play a role in downregulating the expression of *TMS1/ASC*. This finding is similar to the results obtained by Stimson et al[23]. Over expression of *TMS1/ASC *was shown to inhibit cellular proliferation and induce DNA fragmentation which can be blocked by a caspase inhibitor [24]. In addition, forced reduction in *TMS1/ASC *promotes cell survival perhaps in a NFκ-B dependent pathway [25]. This makes it a therapeutic target by use of demethylating agents alone or in combination with additional apoptosis inducing drugs.

ChIP analysis showed binding predominantly with MBD3 and only minimal enrichment of the other MBDs and MeCP2_. _Earlier studies have demonstrated that MBD3 does not bind to methylated DNA alone [26,27]. However, it has a definite role in maintaining methylation. Recent studies have shown binding of MBD3 to several genes like cox6c, leng6, bat5 etc. [28]. Wade et al showed that MBD3 is a subunit of the NuRD complex that has nucleosome remodeling and histone deacetylase activities [29]. MBD3 forms a part of the multiprotein NuRD complex and probably has a role as a transcriptional co-repressor. Our finding that MBD3 binds to methylated *TMS1/ASC *is contrary to the known pattern of MBD binding by MBD proteins to methylated DNA. This could be because MBD3-containing NuRD complexes bind more specifically to the methylated *TMS1/ASC *gene [28].

A noteworthy finding in our study was the statistically significant difference in the methylation of *TMS1/ASC *among the cases and controls in Whites compared to Blacks. We observed an age adjusted odds-ratio of 7.6 (95% CI 2.1–27.3) in Whites as compared to only 1.1 (95% CI 0.2–5.5) among Blacks. Whether this finding reflects involvement of different pathogenetic pathways in different races will be interesting to study. In the US, the incidence and mortality of prostate cancer is about two-fold higher among Blacks compared to Whites, suggesting racial differences in prostate tumor occurrence and aggressiveness [30]. The reason for these racial differences is not well understood. It is possible that promoter-region gene hypermethylation may be influenced by environmental exposures.

There have been only a few studies on differences in gene methylation between different races. Two studies by Woodson et al showed a differential methylation pattern and expression of CD44 in Blacks and Whites [31,32]. When we examined 5 genes frequently methylated in prostate cancer (GSTP1, CD44, ECAD, RASSF1A and EBR) in the same patient population we did not find any significant differences in the gene specific methylation pattern between the different races (Table 5). Ethnic group related differences in hypermethylation of promoter region of GSTP1 gene were reported in a recent paper [33]. The authors observed higher hazard ratio (HR) for pathogenesis among African Americans as compared to Caucasians. In contrast to the above observation, we found the hazard ratio of *TMS1/ASC *methylation (prostate cancer versus BPH) to be lower in Blacks as compared to Whites. Ethnic origin is an important determinant of prostate cancer risk, incidence, and disease progression. In the US, the African-American male group has the highest incidence rate for prostate cancer [30]. Differences in diet, socioeconomic environment, lifestyle between the two ethnic groups have been implicated as causative factors for the striking ethnic differences in the incidence and clinical behavior of prostate cancer. However, molecular mechanisms underlying the racial diversity are not well understood. The recent report by Fang et. al., shows that Genistein leads to reversal of hypermethylation and reactivation of p16INK4a, RARβ, and MGMT genes [34]. Thus, diet seems to be an important factor in affecting the methylation status of different genes implicated in cancer. It is also likely that genes are differentially methylated in different ethnic groups owing to the lifestyle and dietary differences. Methylation of promoter in controls (BPH) may reflect that epigenetic alteration of the gene has already occurred and that they have acquired epigenetic malignant potential even though the pathological diagnosis classifies them as benign [33]. Our results indicate that differences in methylation pattern of *TMS1/ASC *in BPH among ethnic groups might explain the differences among different racial groups in susceptibility to prostate cancer. Thus it seems that the epigenetic make up of different ethnic groups would determine the risk to prostate cancer pathogenesis.

**Table 5:** Risk of prostate cancer in relation to gene methylation, total patients and by race.

	**Number and (%) of tissues with gene methylation**		**Age-adjusted**	
**Gene**	**Prostate Cancer**	**Control (BPH)**	odds ratio* (95% CI)	**P-value**
**TMS1/ASC**	42/66 (63.6)	12/34 (35.3)	3.7 (1.4 – 9.4)	0.008
Blacks	14/21 (66.7)	7/12 (58.3)	1.1 (0.2 – 5.5)	0.914
Whites	28/45 (62.2)	5/22 (22.7)	7.6 (2.1 – 27.3)	0.002
**GSTP1**	46/66 (69.7)	1/34 (2.9)	69.7 (8.7 – 558.7)	<0.0001
Blacks	15/21 (71.4)	0/12 (0.0)	--	
Whites	31/45 (68.9)	1/22 (4.6)	--	
**CD44**	46/66 (69.7)	13/34 (38.2)	2.7 (1.1 – 6.9)	0.033
Blacks	14/21 (66.7)	4/12 (33.3)	2.9 (0.6 – 14.4)	0.190
Whites	32/45 (71.1)	9/22 (40.9)	2.6 (0.9 – 8.1)	0.093
**ECAD**	39/66 (59.1)	5/34 (14.7)	7.9 (2.6 – 24.0)	<0.001
Blacks	15/21 (71.4)	2/12 (16.7)	10.9 (1.7 – 70.9)	0.013
Whites	24/45 (53.3)	3/22 (13.6)	7.2 (1.8 – 29.2)	0.006
**RASSF1A**	34/66 (51.5)	6/34 (17.7)	4.1 (1.5 – 11.8)	0.008
Blacks	10/21 (47.6)	1/12 (8.3)	8.6 (0.9 – 84.7)	0.064
Whites	24/45 (53.3)	5/22 (22.7)	3.2 (0.9 – 10.7)	0.056
**EBR**	49/66 (74.2)	23/34 (67.7)	1.3 (0.5 – 3.5)	0.582
Blacks	15/21 (71.4)	7/12 (58.3)	1.9 (0.4 – 9.5)	0.460
Whites	34/45 (75.6)	16/22 (72.7)	1.1 (0.3 – 3.7)	0.912

* For the overall odds ratio estimate, we modeled the logit of the probability of being a case (p), as a function of methylation in the particular gene and age: ln [p/(1-p)] = b_0 _+ b_1 _× gene + b_2 _× Age. For the race-specific age-adjusted odds-ratio estimates, the following model was fitted: ln [p/(1-p)] = b_0 _+ b_1 _× gene + b_2 _× Age + b_3 _× Race + b_4 _× gene × Race. The interaction between TMS1/ASC and race was marginally significant (p = 0.068), while the interactions between each other genes and race were not statistically significant at the 5% level (that is, corresponding p-values were greater than 0.454).

There did not appear to be a significant relationship between *TMS1/ASC *methylation status and patient's age. On our limited dataset there was a trend towards association between *TMS1/ASC *methylation and Gleason score 7 or higher (odds-ratio 2.3). Though the relationship was not statistically significant it possibly suggests a worse prognosis as patients with Gleason score 7 do worse that those with Gleason score 6. Many of our patient samples showed partial methylation status – this could be due to presence of normal fibroblasts, endothelial cells, inflammatory cells and non malignant prostate tissue surrounding the tumor. Tumors where *TMS1/ASC *was not found to be methylated could involve other genes in the apoptotic pathway. Use of pathway specific cDNA microarrays may help in determining the individual genes affected.

In summary our study has shown that *TMS1/ASC*, a pro-apoptotic gene, is silenced by hypermethylation of the CpG islands in the promoter region. This transcriptional repression is relieved by treatment with a demethylating agent (5-Aza-2'-deoxycytidine). Frequent methylation of *TMS1/ASC *in prostate cancer suggests this gene may be important in pathogenesis of prostate cancer and can be a target of pharmacologic demethylation in clinical trials. *TMS1/ASC *methylation patterns show significant ethnic differences. Our findings provide a novel insight into the molecular determinants of tumor growth that may underlie the ethnic differences in prostate cancer incidence and clinical behavior. Further studies are needed to find out if this has any significant clinical implications in the development of novel diagnostic approaches for biologically aggressive prostate cancer from diverse racial origin.

## Methods

### Cell lines

LNCaP, PC3 and DU145 prostate cancer cells, obtained from American Type Culture Collection were maintained in DMEM (Invitrogen, Carlsbad, CA) and supplemented with 10% fetal bovine serum in a humidified incubator at 37°C with an atmosphere of 5% CO_2_.

### Tissue samples

This IRB-approved study involved patients who either had radical prostatectomy between 1998 and 2002 for prostate cancer or trans-urethral resection between 2000 and 2002 for benign prostatic hypertrophy. Sixty-six prostate cancer and 34 benign prostatic hyperplasia (BPH) patients were selected for the study. The prostate-cancer cases and the BPH controls represented all patients who had archived tissue available for study. The characteristics of these two groups are shown in Table 2. The Whites were of northern European descent and did not include Hispanics

### Drug treatment of prostate cancer cell lines

For 5-Aza-2'-deoxycytidine (Sigma, St. Louis, MO) and Trichostatin A (TSA) (Sigma St. Louis, MO) treatment, LNCaP cells were grown in 100 mm dishes and treated with 1 μm 5-Aza-2'-deoxycytidine and 300 nm TSA. 5-Aza-2'-deoxycytidine treatment was continued for 7 consecutive days where as TSA treatment was done for 6 hours on the final day. RNA, DNA and chromatin were extracted on day 7.

### RNA extraction and reverse transcriptase PCR (RT-PCR)

RNA was extracted from the cell lines using RNA Stat 60 (Tel-Test Inc. Friendswood, TX) as per the manufacturer's instructions. cDNA was prepared from 10 μg of RNA using the Reverse Transcription System from Promega (Madison, WI) using random primers. Two μl of the cDNA was used for the PCR reaction. The primers and PCR conditions used are mentioned in Table 1. Human beta actin (ACTB) was used as the housekeeping gene for loading control.

### DNA extraction, bisulfite treatment and methylation-specific PCR (MS-PCR)

Methylation patterns of the prostate cancer cell lines and the tissue samples were analyzed by MS-PCR of bisulfite treated DNA. DNA was extracted from the prostate cancer cell lines using DNA Stat 60 (Tel-Test Inc. Friendswood, TX.). DNA from archived paraffin blocks was isolated using a QIAmp mini-kit (QIAGEN, CA) as per the manufacturer's protocol. This method successfully isolates DNA suitable for PCR amplification from fixed tissues. Genomic DNA was treated with sodium bisulfite under conditions that converts unmethylated cytosine to uracil while the 5-methylcytosine remains unchanged [35,36]. The bisulfite conversion reaction was carried out by incubating 5 μg DNA with a 5 M bisulfite solution and 100 mM hydroquinone, pH 5.0 at 50°C for 4 hours. This was followed by desulfonation by addition of 3 M NaOH, and desalting using a QIAquick column (Qiagen, CA). MS-PCR was performed using methylated and unmethylated *TMS1/ASC *primers (Accession No. AF184072). The primers described by Virmani et. al., [15] located in the promoter region were used in the study. Details of the primers used are mentioned in Table 1. Human placental DNA was methylated in vitro using *sssI *DNA Methylase (NEB) and bisulfite treated for use as a positive control.

### Bisulfite sequencing

Five μl of the bisulfite treated DNA from LNCaP, PC3 and Du145 cell lines was amplified by PCR using the BST sequencing primers. The primers were designed to amplify both methylated as well as unmethylated DNA. The PCR products were run on a 2% agarose gel and the desired band was gel purified using Promega SV gel purification Kit (Promega Corp). The Thermo Sequenase Radiolabeled Terminator Cycle Sequencing Kit (USB Corp., Cleveland, OH) was used for sequencing using forward and reverse primers. The sequencing gel was dried, and radioactive bands were analyzed using Storm phosphor imager (GE Health Care).

### Chromatin Immunoprecipitation (CHIP) and Real time PCR

Chromatin immunoprecipitation assays were carried out with a kit from Upstate Biotechnology (Lake Placid, NY) using the manufacturer's protocol and reagents except that the reactions were scaled down ten-fold. Briefly, 2 × 10^7 ^cells were incubated in 0.5% formaldehyde for ten minutes to crosslink bound proteins, washed, lysed in SDS lysis buffer and sonicated to 100–500 bp lengths. Ten μl chromatin was mixed with 90 μl of ChIP dilution buffer and precleared with Protein A agarose, and then the chromatin was incubated with anti acetylated H3 and H4 antibody overnight at 4°C. Thirty μl of Protein A agarose beads was added and the chromatin was immunoprecipitated 2 hours at 4°C. The supernatant (unbound chromatin) and beads (bound chromatin) were separated. The beads were washed five times with the buffers provided and then the chromatin was eluted twice in 1%SDS in 0.1 M NaHCO_3_. Cross-linking was reversed by adding 5 M NaCl and incubating at 65°C for at least 4 hours, following which proteinase K digestion was carried out and DNA was extracted with phenol/chloroform. DNA was ethanol precipitated and dissolved in 100 μl of water.

Real time PCR was performed in triplicate using 5 μl of the immunoprecipitated DNA using primers for *TMS1/ASC *and GAPDH as a housekeeping gene. Quantitative PCR was carried out in a reaction volume of 25 μl using iQ™ SYBR^® ^Green Supermix (BioRad Laboratories, Hercules, CA) on MyiQ™ Single-Color Real-Time PCR Detection System (BioRad Laboratories, Hercules, CA). The final reaction mixture contained 400 nmol/L of each primer. PCR was done for 50 cycles at an annealing temperature of 68°C for *TMS1/ASC *and 65°C for GAPDH. A standard curve was prepared for both the genes using a serial dilution of sonicated human placental DNA (Sigma Chemical Co.). Appropriate negative controls were included in each run. Ratio of *TMS1/ASC *to GAPDH was calculated for each sample.

### Statistical analyses

SAS software (release 8.2, SAS Institute) was used for statistical analyses. Student's t test (Armitage, Berry & Matthews 2001) was used to compare the average age in the BPH and prostate-cancer groups. Logistic regression (Hosmer & Lemeshow, 2000) was used to determine the effect of methylation in the particular gene on prostate cancer risk, whether this effect was similar for Blacks and Whites, and whether *TMS1/ASC *gene methylation was associated with Gleason score, clinical stage, or age of prostate cancer onset. In particular, since prostatic tumors with Gleason-score values of 7 have a substantially worse clinical course than those with values of Gleason score 6, and since few patients (only 10%) had Gleason-score values other than 6 or 7, we used logistic regression to compare the relative frequency of *TMS1/ASC *methylation in tumors with Gleason score 7 versus 6. For association with clinical stage, we likewise compared *TMS1/ASC *methylation in stage 3 versus stage 2 tumors because these two stages comprised 93% of tumors studied. Since prostate-cancer patients were on average 7 years younger than the BPH controls, all of the odds-ratio estimates of relative risk associated with a particular gene methylation were adjusted for age as a continuous covariate in the logistic regression analyses.
